# Supplementary material for: Asynchronous magnetic resonance elastography: Shear wave speed reconstruction using noise correlation of incoherent waves
Source: Magn Reson Med. 2022 Oct 27;89(3):990–1001. doi: 10.1002/mrm.29502 (PMC9792433; doi:10.1002/mrm.29502)
Supplement: Supplementary file 1 — DATA S1 MATLAB code used to generate the numerical simulations of Figure 1 [file MRM-89-990-s002.zip › k-Wave/helpfiles/scaleSI.html]

scaleSI :: Functions (k-Wave)


# scaleSI

Scale a number to nearest SI unit prefix.

## Syntax

```
x_sc = scaleSI(x)
[x_sc, scale] = scaleSI(x)
[x_sc, scale, prefix] = scaleSI(x)
[x_sc, scale, prefix, prefix_fullname] = scaleSI(x)
```

## Description

`scaleSI` scales the input `x` to use the nearest SI unit prefix while keeping 1000 > x > 1. For example, `scaleSI(0.00001)` would give `x_sc = '10u'`, `scale = 1e6`, `prefix = 'u'`, and `prefix_fullname = 'micro'`.

## Inputs

|  |  |
| --- | --- |
| `x` | number to scale |

## Outputs

|  |  |
| --- | --- |
| `x_sc` | string of scaled input and prefix |
| `scale` | numeric scale factor |
| `prefix` | single character scale prefix |
| `prefix_fullname` | full SI name for prefix |

## Examples

- Recording The Particle Velocity
- Defining A Gaussian Sensor Frequency Response
- Comparison Of Modelling Functions
- Photoacoustic Waveforms in 1D, 2D and 3D
- Monopole Point Source In A Homogeneous Propagation Medium
- Dipole Point Source In A Homogeneous Propagation Medium
- Steering A Linear Array
- Simulations In Three-Dimensions
- Focussed Detector in 2D
- Image Reconstruction With Compensation For Acoustic Absorption
- Source Smoothing
- Modelling Power Law Absorption

## See Also

`scaleTime`
